# Using a route planner to optimize door-to-door visits for a pediatric home-hospitalization program: Feasibility study

**DOI:** 10.3389/fped.2022.928273

**Published:** 2022-07-22

**Authors:** Astrid Batlle, Imma Boada, Santiago Thió-Henestrosa, Mariona Fernández de Sevilla, Juan José García-García

**Affiliations:** ^1^Hospital Sant Joan de Déu, Barcelona, Spain; ^2^Graphics and Imaging Laboratory, University of Girona, Girona, Spain; ^3^Departament Informàtica, Matemàtica Aplicada i Estadística, University of Girona, Girona, Spain

**Keywords:** home-hospitalization, door-to-door, vehicle routing problem (VRP), route planner application, environmental costs, optimization algorithm

## Abstract

**Background:**

Hospital Sant Joan de Déu (Barcelona) initiated a pediatric acute home-hospitalization program. Due to high patient turnover and the health staff's lack of planning training, daily scheduling was a time-consuming task. Home-hospitalization planning is a vehicle routing problem that can be solved with a technological solution. It was therefore decided to evaluate the efficacy and necessity of the SmartMonkey.io planner.

**Objectives:**

To compare traditional manual route planning with a route optimizer, and to evaluate the technical feasibility of the implementation of a route planner into a homecare program.

**Methods:**

Eight participants (experienced homecare staff and inexperienced hospital staff) were included. Personal interviews were performed to assess their eagerness to try a technological solution to the planning problem. Objective benefits including reduced travel time (time planning, distance traveled, and time traveled) were evaluated. Paired *t*-test, *t*-test, and Pearson's correlation were used to compare manual and route planner scheduling. Participants then answered a questionnaire to assess planning difficulty and the acceptance of the route planner.

**Results:**

Homecare staff were initially reluctant to use the technology. Significant differences (*P* < 0.0001) in three variables were found between manual planning and the route planner. A moderate correlation between time planning and plan difficulty (*r* = 0.59, *P* < 0.0001) was found with manual planning but not with the route planner. All route planner schedules saved time and distance. No significant differences were found between expertise and planning method. It was noted that it was easy to create plans with the route planner, while difficulty with manual planning increased as more locations were added. All participants evaluated the route planning tool favorably.

**Conclusions:**

Route-planning technology saved planning time and generated better plans than manual planning. The route planner's learning curve was fast and results were obtained in the same amount of time regardless of difficulty and expertise. SmartMonkey.io also has the potential to reduce internal and environmental costs and increase staff productivity.

## Introduction

The demand for homecare has grown rapidly over the last decade due to high healthcare costs and fewer available hospital beds, the result of the increased prevalence of chronic diseases that require abundant resources (1). Home hospitalization is a good alternative to inpatient hospitalization for eligible patients. While experiences with home hospitalization of adults are well-documented, there are fewer pediatric experiences, most of which focus on chronic and palliative pediatric patients. Nevertheless, over the past few years pediatric homecare has also become a good alternative to hospitalization for acutely ill children.

Homecare enables patients to feel more comfortable in their environment, and prior literature has shown that it has high levels of acceptance by families and children (2). Homecare has high levels of safety and quality compared with inpatient hospitalization (3, 4) and urges healthcare professionals to be more attentive to the needs of the patient and their family, allowing them to participate more in care (5). Homecare takes into consideration all four pillars of medical ethics (beneficence, non-maleficence, autonomy, and justice), especially autonomy, by giving patients and families the freedom to choose and take care of their children if they are able. In fact, in cases of patients with complex chronic conditions, an infant's caregivers are well-prepared as they have taken care of their own children for years (5). Patients treated at home also feel more supported and report improved quality-of-life (5).

Homecare reduces costs compared with inpatient hospitalization (3, 6). Cost reduction has been driven by lower levels of healthcare utilization and, in some cases, decreased length of stay (7). It has also been suggested that home hospitalization programs reduce 30-day readmissions (3, 8). This is likely because of better discharge planning, as caregivers would have been performing tasks in-house that could now be done in the patient's home (3). Hospitalization at home frees up hospital beds for more complex and unstable patients, making the health system more sustainable (9).

The main characteristics of acute homecare are: (1) short-term, limited admissions; (2) complex care, because if home hospitalization did not exist the patient would be admitted to hospital; (3) efficacy, attending to appropriate care and patient satisfaction; and (4) sustainable use of healthcare resources (10). Two characteristics make pediatric acute hospitalization at home different from that of adults. The first is that children are normally healthy individuals with few comorbidities or poly-pharmacies, leading to shorter hospital admissions. The second is that caregivers, usually the children's parents, are younger and eager to learn and understand new challenges. These two features imply that pediatric in-home hospitalizations will have a high patient turnover and there will be increased eagerness for caregivers to take care of their children and use new technology.

In this context, in April 2019 an acute hospitalization at home program was initiated in Hospital Sant Joan de Déu in Barcelona. This is a tertiary hospital with more than 25.000 discharges, ~238.000 outpatient visits, and 122.000 emergency visits per year. It receives complex national and international patients, and also admits patients of low complexity from the territory. In our center, pediatric patients are admitted to hospital at home from the inpatient ward, emergency department, or outpatient department. The patient's inclusion is voluntary. Inclusion criteria are: (1) patients from 0 to 18 years old with an acute or chronic exacerbated disease that implies hospital care, (2) living within 30 min of the hospital, (3) a 24-h trained caregiver at home, (4) minimum habitability conditions at home; and (5) the possibility of telephone contact if necessary. Exclusion criteria are a language barrier that prevented empowerment of the caregiver. The main pathologies that are treated at home are infectious and respiratory diseases, while the main treatments are outpatient antibiotic therapy, supplemental oxygen therapy, and nebulization. Patient follow-up is performed using telemedicine, face-to-face visits, and remote telemonitoring. After the caregivers are trained on appropriate care for their child (administering treatments, using monitoring dispositive, and recognizing alarm signs) the patient is discharged home for continued hospital care.

Such a program faced some challenges during its first implementation, notably follow-up at home and door-to-door daily planning. The first challenge was solved rapidly thanks to telemonitoring, which had good outcomes for both families and professionals (11). With respect to door-to-door planning, rapid patient turnover made it difficult to manage daily scheduling. Unlike logistics jobs, which incorporate specific training in route planning, the health staff syllabus does not consider these cases. One of the main issues is the health staff's poor knowledge of the surrounding geography, which therefore requires a significant amount of additional daily time for scheduling. Further, as there is no specific training on this matter, once health staff are sufficiently skilled it is difficult to replace them when needed.

Being aware of the door-to-door planning problem, the hospital technology team suggested a technological solution. After studying the market, three solutions were proposed: Beetrack (12), Simpliroute (13), and SmartMonkey.io (14). Different aspects of each tool were considered, such as onboarding ease, features, support service, and price. After the technology team tested all three solutions, SmartMonkey.io was chosen. It was affordable and easy to use, as plan generation took fewer steps compared with other solutions. It also had instructional videos. Nonetheless, as the health care staff was the end-user group, a feasibility study was performed to evaluate the tool and establish the necessity for its implementation.

This trial was focused on the use of the route optimizer SmartMonkey.io to generate hospital-at-home routes. The vehicle routing problem (VRP) has been widely studied, as it is a complex problem (NP-Hard) that can be approached using multiple algorithms. There are some studies in the literature that describe the use of optimizers specifically in homecare, mostly focusing on the mathematical algorithm (15–18). However, no works have described the results of applying real commercialized software for homecare program planning using real locations and constraints, with healthcare staff as participants, nor have such operational procedures been accepted by healthcare teams (19).

Bowen et al. describe eight general areas of focus for a feasibility study, which were evaluated during the trial: (1) participant acceptability; (2) demand for the intervention; (3) implementation; (4) practicality, in terms of resources and participant commitment; (5) adaptation to the new situation; (6) integration of the new process in the actual environment; (7) expansion into another environment; and (8) efficacy testing, both when limited to the study or as applicable to daily work (20).

In our case problem, software evaluation was performed using both qualitative and quantitative data. Quantitative data were extracted from the health staff's real patients' location scheduling. Both quantitative and qualitative data were collected to evaluate the subjective competence and feelings of the participants regarding the tool, following the Technology Acceptance Model (TAM) (21), which is a widely used model that makes it possible to obtain information regarding how users come to accept a technology. It suggests that there are two factors that influence the decision to use a technology: (i) perceived usefulness (PU) and (ii) perceived ease-of-use (PEOU). PU was defined by Davis as “the degree to which a person believes that using a particular system would enhance their job performance”; PEOU is defined as “the degree to which a person believes that using a particular system would be free from effort,” that is, whether it will be easy to use. Prior authors have described prior and contextual factors (22–25) that can model TAM.

The expected outcomes of applying the VRP algorithms were described by Jemai and are: (1) to reduce traveling distance and traveling costs, (2) to improve worker productivity, (3) to increase customer service by satisfying all service requirements, (4) to satisfy employee preferences, and (5) to distribute work equally (26).

The aims of this study are: (i) to compare the performance of traditional manual route planning vs. the route optimizer solution and (ii) to evaluate the technical feasibility of the implementation of route optimization into a home care program.

## Methods

### Case problem

The hospital-at-home staff are comprised of two pediatricians and four nurses, in addition to administrative and technological support. Home visits take place during the morning, 7 days a week (nurse attention only on non-working days), and families have 24-h telephone contact if needed. A health team formed by a pediatrician and a nurse travel daily to each patient's home to check on the child, administer treatments, and solve caregivers' concerns regarding the patient's condition and follow-up.

The maximum capacity of the service is 12 patients (including face-to-face and telematic visits) and the maximum capacity of door-to-door visits is 10 patients. Some patient conditions required home visits at a certain time or by a certain pediatrician. There are normally two different routes per day, conducted by two teams consisting of a pediatrician and a nurse. At times an extra route is needed as some patients require only nursing care.

All route plans are designed by hospital-at-home health staff, either nurses or pediatricians.

The health staff operated with a hospital fleet to perform home visits. The cars used in this program are an Opel Corsa 1.5D DT 74 kW and a Smart EQ ForTwo. The CO2 emissions of the diesel car are ~142 g/km, according to the spec sheet, while that of the electric car are 16.6 kWh/100 km (CO2 emissions must be considered 0.25 kg CO2/kWh due to the production of electricity, based on the Spanish regulator in 2021).

### SmartMonkey planner: The route optimizer

SmartMonkey.io is a company that was founded in Barcelona and operates in 26 countries. It developed a route optimization platform for improving last-mile operation performance for logistics, maintenance, and service companies.

This route optimizer is used by small and medium enterprises and big corporations, but has not been used in home care planning. The software provides an easy-to-use interface for solving the VRP with several constraints, such as: time windows, multiple capacities, business restrictions, traffic, pick-up and delivery, and circular routes. It also integrates real-time tracking of practitioners and customer communication functionalities that are outside the scope of this study.

SmartMonkey's platform is straightforward, and the main user flow can be performed in <10 clicks. It also has an onboarding tutorial and several videos that allow users to understand its different functionalities easily and rapidly.

**Figure 1:**
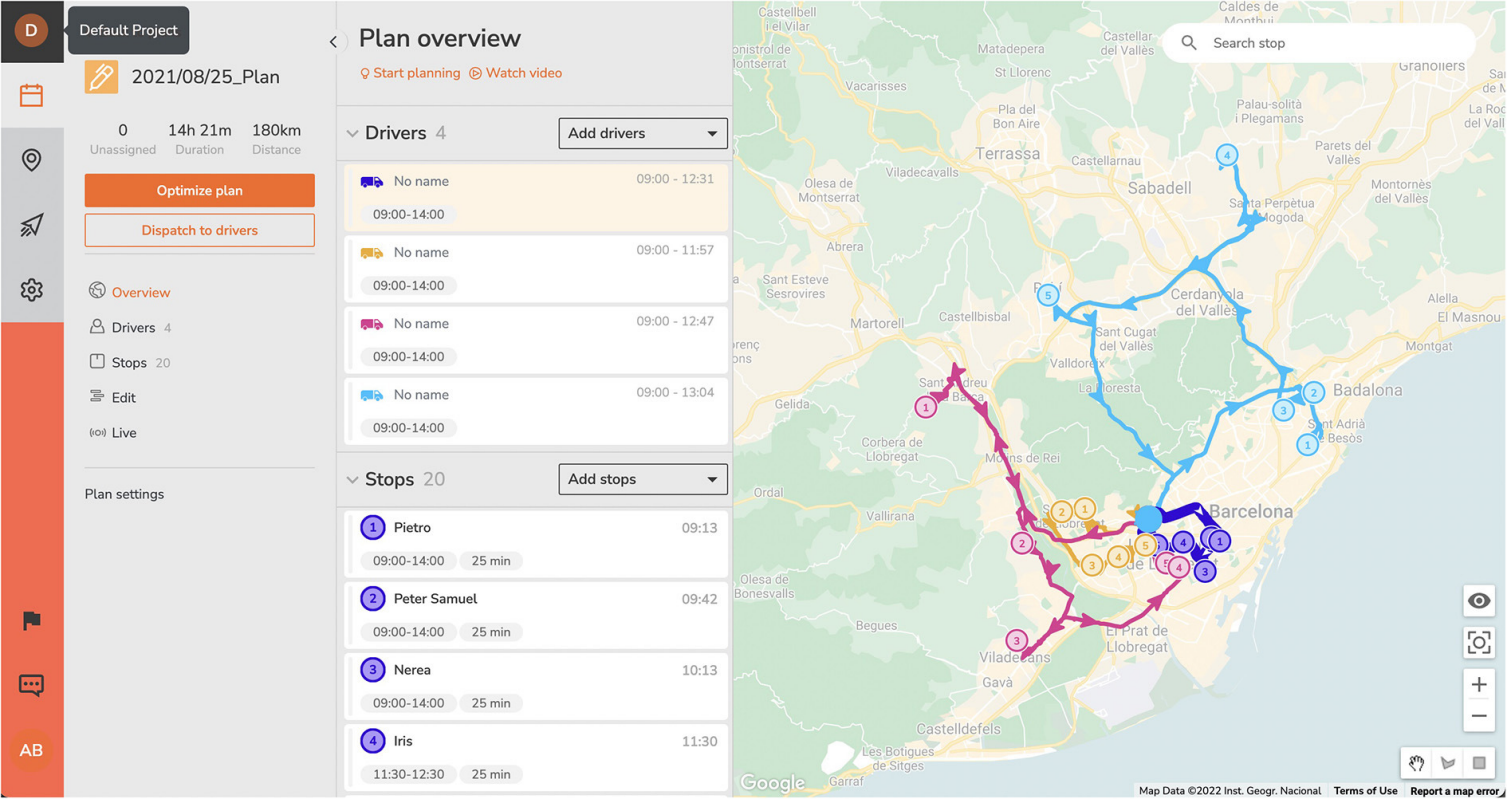
Optimized plan with SmartMonkey.io.

The method followed to optimize each plan was: (1) introduce a spreadsheet file into the platform with the addresses and constraints of each patient. The platform automatically geolocates all positions and shows a map of the territory; (2) introduce the number of available resources, meaning the number of home-healthcare teams and their constraints; (3) obtain the optimized plan in less than a second; and (4) perform manual modifications as needed. The design of an optimized plan can be seen in Figure 1.

### Participants

Test participants comprised eight subjects: four nurses and four pediatricians. These were divided into two groups: (a) experienced hospital-at-home staff and (b) inexperienced hospital staff. The first group was formed by personnel working in the hospital-at-home program of Hospital Sant Joan de Déu and included two nurses and two pediatricians who had already performed manual route planning. The second group included personnel working at Hospital Sant Joan de Déu not related to the hospital-at-home program and included two nurses and two pediatricians who had never been involved in route planning.

The two groups received a 30-min training session on the basic use of the SmartMonkey.io route planner before using it.

All the participants agreed to participate in the study voluntarily. The experiment was conducted according to the principles of the Declaration of Helsinki.

### Experimental design

To understand the trial, we defined the following: (a) a plan is a group of routes in a single day and (b) a route is a group of locations in a set order with some constraints.

The following constraints were considered: (1) route time windows that determine the working time of each team; (2) patient time windows, which are the times during which the patient must be seen; (3) the number of routes; and (4) specific health staff requirements per patient, understanding that some patients require only a nursing visit and others might require a pediatrician and a nurse.

All the locations met the criteria of 30 min isochron from the hospital and 25 min per patient visited.

Participants were allowed to make manual adjustments in the route planner preliminary results. Due to the ambiguity of the constraints, these may be introduced into the platform differently. For example, in our case problem, when a time window is required to limit working hours (while a team can work the whole day, another one only works half the hours). This constraint could be introduced into the software by limiting the time windows or the number of stops per vehicle. This would generate different results that may or may not be convenient to each participant based on their work and territorial experience and that may require a final manual adjustment. As a result, travel time and distance differences between the participant planned routes are expected.

Note that although all of the locations and constraints are real, the plans that the participants must schedule were not executed.

The experiment was performed in three phases.

#### Phase 1

The objective of Phase 1 was to determine if the nurses and pediatricians that worked in the program were eager to try a technology solution for the planning problem. To acquire information on this question, individual interviews were performed among the home hospitalization staff. Workers were interrogated using the TAM. Questions were aimed to determine: (1) PU, in particular the ease of manual route planning, if the participant liked it or not, and the need to find another way to solve the problem; and (2) prior and contextual factors that could interfere with the use of the technological tool, including questions about the participant's eagerness to implement a new technology in their daily work to solve the route planning problem.

#### Phase 2

The objective function of a route planner is to reduce travel time. All constraints must be satisfied, and all locations must be scheduled. Ten plans based on the homes of real patients were selected. Participants were asked to schedule the same plans, first manually and then with the route planner. Five of the plans had 10 locations per plan, while three plans had 15 locations and two plans had 20 locations. Every plan had one or more of the previously described constraints (Table 1).

**Table 1:** Plans' specifications and their constraints.

**Plan**	**Description**
1	10 locations in 2 routes, the same time window each.
2	10 locations in 3 routes, different time windows per route.
3	10 locations in 2 routes, the same time window each + 1 patient time window.
4	10 locations in 3 routes, different time windows per route + 1 patient time window.
5	10 locations in 2 routes, 2 locations with specific health care team required.
6	15 locations in 3 routes, the same time window each.
7	15 locations in 3 routes, the same time window each + 1 patient time window.
8	15 locations in 3 routes, the same time windows each + 2 locations with specific health care team required.
9	20 locations in 4 routes, the same time window each.
10	20 locations in 4 routes, the same time window each + 1 patient time window.

Plan difficulty was defined by the number of locations per plan. The complexity of each plan was assumed to be the number of possible solutions (n!, where n is the number of locations). Although not quantified, constraints also modified the complexity of the plan in some manner. The more constraints, the more difficult the plan.

Three grades of difficulty were defined: (a) easy, defined as 10 location plans; (b) medium, for 15 location plans; and (c) hard, for 20 location plans.

Distance and travel time were estimated using the SmartMonkey.io planning tool. In the specific case of manual planning, the results given by each participant were introduced manually to the platform in order to measure distance and travel time.

#### Phase 3

All participants (home hospitalization staff and non-home hospitalization staff) were asked to answer a multiple-choice questionnaire about PU and PEOU after using the route optimizer. Questions were aimed at assessing the difficulty of planning routes (PU) and the acceptance, usability, and convenience of the route planner (PEOU) (Table 2).

**Table 2:** Phase 3: questionnaire to evaluate the usability and convenience of the route planner.

**Difficulty on planning routes (Perceived usefulness) (1 = totally disagree, to 5 = totally agree)**
Q1. It was easy for me to create the 10 locations plan manually.
Q2. It was easy for me to create the 20 locations plan manually.
Q3. It was easy for me to create the 10 locations plan with the route planner.
Q4. It was easy for me to create the 20 locations plan with the route planner.
**Usability (Perceived ease-of-use) (1 = totally disagree, to 5 = totally agree)**
Q5. It was easy for me to understand how to create plans with the route planner.
Q6. The speed to get results was fast.
**Global evaluation of the route planner (Perceived Ease-of-Use) (1 = totally disagree, to 5 = totally agree)**
Q7. Globally, I favorably evaluate the route planner.
Q8. Globally, I would recommend other hospital-at-home teams to use the route planner.
Q9. Globally, I think it is convenient to have the route planner to facilitate daily work.

### Statistical analysis

A paired *t*-test was used to compare the time planned, distance traveled, and time traveled between the manual and route planner schedules. We assume that when analyzing the difference between times, all differences can be considered independent, that is, not dependent on the eight participants who performed the analysis. Paired *t*-test was also used to compare the same variables when stratified by difficulty. A *t*-test was used to compare the two groups of expertise, either with manual scheduling or route planner scheduling. A Pearson's correlation coefficient between time planning and plan difficulty was calculated.

The Mann-Whitney *U* test was used to compare the questionnaire results between the experienced group and the inexperienced group. R software was used in all cases (27).

## Results

### Phase 1: Individual interviews before tool implementation

Individual interviews were carried out with four hospital-at-home workers. In terms of PU, when asked about their experience with manual route planning the average experience was 7.7 months (two of the staff members had been working in home hospital care for 1 year, while the other two had been working there for 3 and 4 months, respectively). With respect to difficulty with planning routes, the staff agreed that the difficulty grew when more patients had to be visited. Two of the workers thought planning <10 points was easy, while difficulties were encountered when planning for more than 10 points. The other two participants thought that any number of points planned manually was a difficult enough task. All participants agreed that route planning was an inconvenience, as they were not trained to do it. They also noted that it would be helpful to have an automatic planning tool to facilitate everyday work.

Asked about the implementation of new software in their daily work, all participants were reluctant to use a new tool, as they already had bad experiences with the implementation of new technology at their hospital. They thought that it would be difficult to cope with any problems that could appear when using the new tool. Some of the staff assertions were: “I feel alone when I have problems with technology at the hospital,” “I am very bad using new technology, I don't understand it,” and “These kinds of technologies need time to be understood, which I don't have.”

### Phase 2: Analyzing quantitative results

The analyzed variables were: (1) planning time, defined as the time needed to schedule a plan; (2) distance, defined as the sum of the consecutive distances of each planned route based on the order defined by the participants; and (3) travel time, defined as the theoretical sum of the travel times of each plan based on the order defined by the participants.

The plans were first divided into two groups by planning method: either manual scheduling (MS) or route planner scheduling (RPS). Variables were then compared between each group. Significant differences (*P* < 0.05) were identified in the three variables, with significant reductions in planning time, distance, and travel time using the RPS method (Table 3).

**Table 3:** Comparing the planning method: MS vs. RPS.

	**Total (*n* = 160)**	**MS (*n* = 80)**	**RPS (*n* = 80)**	***P*** **(paired *t* test)**
**Planning method**				
Planning time (min) (mean;sd)	7.52; 5.42	11.23; 5.22	3.81; 2.03	<0.0001
Distance (Km) (mean; sd)	117.41; 44.91	119.82; 47.20	115.01; 42.66	<0.0001
Travel time (min) (mean; sd)	539.73; 162.05	545.04; 164.66	534.42; 160.26	<0.0001

*Mean and standard deviation (SD)*.

Each group (MS and RPS) was also analyzed by route difficulty (easy, medium, and hard). Significant differences were seen in all the cases except for the distance of medium-difficulty plans (Table 4). A moderate correlation was found between time planning and plan difficulty for the MS method (*r* = 0.59, *P* < 0.0001), but not the RPS method (*r* = −0.12, *P* = 0.2875).

**Table 4:** Comparing the planning method attending to difficulty.

**Plan difficulty: Easy. comparing planning method**				
	**Total (*n* = 80)**	**MS (*n* = 40)**	**RPS (*n* = 40)**	***P*** **(paired *t* test)**
Planning time (min) (mean; sd)	6.45; 3.63	8.78; 3.32	4.11; 2.10	<0.0001
Distance (Km) (mean; sd)	84.43; 18.60	85.67; 18.41	83.20; 18.95	0.002
Travel time (min) (mean; sd)	402.55; 29.57	406.75; 27.90	398.35; 30.93	<0.0001
**Plan difficulty: Medium. comparing planning method**				
	**Total (*n* = 48)**	**MS (*n* = 24)**	**RPS (*n* = 24)**	**Valor *P* (paired *t* test)**
Planning time (min) (mean; sd)	7.45; 4.79	11.26; 3.62	3.64; 1.85	<0.0001
Distance (Km) (mean; sd)	121.40; 17.36	122.40; 18.17	120.40; 16.85	0.358
Travel time (min) (mean; sd)	582.62; 17.36	586.04; 17.14	578.21; 16.92	0.033
**Plan difficulty: Hard. comparing planning method**				
	**Total (*n* = 32)**	**MS (*n* = 16)**	**RPS (*n* = 16)**	***P*** **-value (paired *t* test)**
Planning time (min) (mean; sd)	10.32; 8.45	17.31; 6.25	3.33; 2.10	<0.0001
Distance (Km) (mean; sd)	193.88; 11.75	201.31; 11.91	186.44; 5.05	0.0007
Travel time (min) (mean; sd)	818.34; 21.62	829.25; 21.45	807.44; 15.87	0.001

*sd, Mean and standard deviation*.

Mean differences between the MS and RPS methods for the three variables were calculated and stratified by difficulty group (Table 5). The average total saved time (planning time plus travel time) per difficulty group ranged from 13.07 to 35.79 min. All RPS plans saved distance, which translates into lower CO2 emissions. Considering the specific cars used in this program, the range of saved CO2 emissions varied by the locations per plan and the vehicle that was used. For the diesel car, emissions varied from 0.10 to 0.77 TCO2 per year; for the electric car, emissions varied from 0.03 to 0.22 TCO2 per year.

**Table 5:** Mean differences between MS method and RPS method.

**Plan difficulty: Easy**	
MS Planning time – RPS Planning time (min)	4.67
MS Distance – RPS Distance (Km)	2.47
MS Travel time – RPS Travel time (min)	8.4
**Plan difficulty: Medium**	
MS Planning time – RPS Planning time (min)	7,62
MS Distance – RPS Distance (Km)	2
MS Travel time – RPS Travel time (min)	7,83
**Plan difficulty: Hard**	
MS Planning time – RPS Planning time (min)	13,98
MS Distance – RPS Distance (Km)	14,87
MS Travel time – RPS Travel time (min)	21,81

When the two groups of participants were compared by expertise, no significant differences were found in the analyzed variables in the MS or RPS method (Tables 6, 7).

**Table 6:** Manual scheduling method: comparing groups attending to expertise.

	Total (*n* = 80)	Experienced (*n* = 40)	Non-experienced (*n* = 40)	***P*** **(*t* test)**
**Manual scheduling method**				
Planning time (min) (mean; sd)	11.23; 5.21	11.97; 5.39	10.50; 5.00	0.210
Distance (Km) (mean; sd)	119.82; 47.20	118.5; 46.49	121.13; 48.45	0.805
Travel time (min) (mean; sd)	545.04; 164.66	542.48; 164.81	547.60; 166.57	0.890

*sd, Mean and standard deviation*.

**Table 7:** Route planner scheduling method: comparing groups attending to expertise.

	**Total (*n* = 80)**	**Experienced (*n* = 40)**	**Non-experienced (*n* = 40)**	***P*** **(*t* test)**
**Route planner scheduling method**				
Planning time (min) (mean; sd)	3.81; 2.03	4.00; 2.29	3.62; 1.73	0.410
Distance (Km) (mean; sd)	115.01; 42.66	115.03; 43.02	114.98; 42.84	0.996
Travel time (min) (mean; sd)	534.42; 160.26	533.80; 164.76	535.05; 157.73	0.972

*sd, Mean and standard deviation*.

### Phase 3: Analyzing usability and convenience

When analyzing the questionnaire (Table 8), it has been shown that it is easy enough to plan for 10 locations manually but difficult to schedule 20 locations per plan. In contrast, it was easy to create 10 or 20 location plans with the route planner. A high PU is therefore demonstrated.

**Table 8:** Analysis of the questionnaire: comparing expertise.

	**Total (*n* = 8)**	**Expert (*n* = 4)**	**Non-expert (*n* = 4)**	***P*** **(*U* Mann-Whitney)**
**Questionnaire: comparing expertise**				
Q1 (Md; IQR)	3.5; 0.93	3.75; 0.5	3.25; 1.26	0.448
Q2 (Md; IQR)	1.625; 1.19	1.5; 1	1.75; 1.5	1
Q3 (Md; IQR)	4.875; 0.35	5; 0	4.75; 0.5	0.453
Q4 (Md; IQR)	4.625; 0.52	4.5; 0.58	4.75; 0.5	0.608
Q5 (Md; IQR)	4.5; 0.76	5; 0	4; 0.82	0.067
Q6 (Md; IQR)	4.5; 0.76	4.25; 0.96	4.75; 0.5	0.505
Q7 (Md; IQR)	5; 0	5; 0	5; 0	–
Q8 (Md; IQR)	5; 0	5; 0	5; 0	–
Q9 (Md; IQR)	5; 0	5; 0	5; 0	–

*Md, Median; IQR, Interquartile Range*.

With respect to usability, it was easy and quick for the participants to create plans with the route planner. It is noted that all participants evaluated the route planner favorably (5/5), thought that it was convenient (5/5), and would recommend that other hospital-at-home teams use it (5/5). A high PEOU was noted globally.

When comparing the questionnaire results by expertise (experienced staff vs. non-experienced staff), no significant differences were found.

## Discussion

### Comparison with prior work

There are multiple articles in the literature that refer to the VRP in home healthcare (28), but all focused on the algorithms and quantitative improvements. When attempting to solve a planning problem for home hospitalization it is essential to not only focus on quantitative improvement, but also on its implementation in a specific environment. In the first phase of this study, it was noted that health caregivers were reluctant to introduce new technology into their daily work. This is the reason why in this feasibility study we took into consideration a real environment: specific hospital-at-home constraints, anonymized real location data, health caregivers as participants, and a specific market tool (SmartMonkey.io) to solve the VRP. Note that although final planning was not performed it is not relevant for this feasibility study, as the final time traveled may be modified because of external agents, such as traffic or a change in a patient's condition.

### Principal results and conclusions

When introducing a new tool into a hostile environment due to the reluctance of homecare staff, it is essential to identify prior and contextual factors that may influence the acceptance of that technology. In this specific case, all homecare staff were aware of the route-planning problem and agreed that it was an inconvenient task. Despite this, workers thought implementing a new technology would cause them discomfort rather than benefits. The necessary features that the new tool had to fulfill were ease of use and implementation, flexibility in relation to patient health requirements, and time efficiency.

When analyzing the quantitative results, it was evident that the technology is effective, as it saved planning time and generated better plans than the ones manually created by the participants. It is also important to focus on the learning curve. As more locations were added to the plans, the difficulty of creating these routes grew significantly. In our trial, as difficulty increased, the MS method needed more planning time to create less effective plans, while the RPS method created better results in less time. However, when participants became skilled at using the tool, they generated the difficult routes in the same amount of time or faster than the easy ones.

Unexpectedly, no differences were detected when participants were divided by expertise. It was initially hypothesized that homecare staff, who had previous experience with manual planning, would have more skills and therefore achieve better results. We believe that the staff members were not experienced enough, as they normally planned routes that included 10 locations or fewer.

These findings are interesting, as they demonstrate that inexperienced staff (such as the home-hospitalization clerk) can take care of daily planning with the route optimizer, releasing health staff to take charge of other more important tasks.

We must focus on two of the targets that result from solving this VRP for home hospitalization: minimizing internal costs and diminishing environmental costs (29). With respect to internal costs, the optimization tool permitted reduced demand for expendable material (fuel and fleet's total cost of ownership) and increased staff productivity. When considering the scale of the homecare program, it was noted that from 15 to 20 locations per plan, the total time saved permitted the homecare program to add one more patient to the daily census (considering a 25 min visit per patient on average). With respect to environmental issues, more efficient door-to-door routes allow the program to be more sustainable and emit less CO2 and other pollutants.

## Limitations

The number of locations per plan used for this study were determined by the actual capacity of the program and its expected short-term growth. Further studies should be performed with more locations per plan to calculate the potential savings in even more complex environments, such as those with a larger census or with additional constraints. Moreover, the cost benefits of implementing the tool and the impact reduction on cost per day for home hospitalizations should be analyzed in detail.

This experiment was performed assuming that each visit was for a predefined time, but some patients may require double the allotted time depending on their pathology. It would be interesting to analyze these times properly to be more precise with daily planning and with the care loads of health professionals.

It would also be of interest to study the use of the software as part of the daily work of different hospital areas, taking into consideration functionalities that have not yet been evaluated, such as real-time practitioner tracking and customer communication functionality.

Despite these limitations, we conclude that our feasibility study has been a success, as healthcare staff identified the VRP and accepted the tool, with positive implementation results. The software received excellent global evaluations and participants clearly adopted the technology with ease, achieving good outcomes. Due to the study's successful results the software has already been implemented in the acute homecare program's daily workflow and will soon be implemented by other hospital areas.

## Data availability statement

The raw data supporting the conclusions of this article will be made available by the authors, without undue reservation.

## Ethics statement

The studies involving human participants were reviewed and approved by Hospital Sant Joan de Déu Ethics Commitee. The patients/participants provided their written informed consent to participate in this study.

## Author contributions

AB contributed to the conception and design of the study, organized the database, and wrote the first draft of the manuscript. ST-H performed the statistical analysis. IB, ST-H, MS, and JG-G contributed to manuscript revision. All authors read and approved of the submitted version.

## Funding

This work has been funded in part by Hospital Sant Joan de Déu and by Spanish Government grants (Nr. MICIU PID2019-106426RB-C31).

## Conflict of interest

The authors declare that the study was performed in the absence of any commercial or financial relationships that could be construed as a potential conflict of interest.

## Publisher's note

All claims expressed in this article are solely those of the authors and do not necessarily represent those of their affiliated organizations, or those of the publisher, the editors and the reviewers. Any product that may be evaluated in this article, or claim that may be made by its manufacturer, is not guaranteed or endorsed by the publisher.
